# Mailed Letter Versus Phone Call to Increase Diabetic-Related Retinopathy Screening Engagement by Patients in a Team-Based Primary Care Practice: Prospective, Single-Masked, Randomized Trial

**DOI:** 10.2196/37867

**Published:** 2023-01-11

**Authors:** Vess Stamenova, Megan Nguyen, Nike Onabajo, Rebecca Merritt, Olivera Sutakovic, Kathryn Mossman, Ivy Wong, Lori Ives-Baine, R Sacha Bhatia, Michael H Brent, Onil Bhattacharyya

**Affiliations:** 1 Institute for Health System Solutions and Virtual Care Women's College Hospital Toronto, ON Canada; 2 South Riverdale Community Health Centre Toronto, ON Canada; 3 Donald K Johnson Eye Institute University Health Network Toronto, ON Canada; 4 Hospital for Sick Children Toronto, ON Canada; 5 Department of Ophthalmology and Vision Sciences University of Toronto Toronto, ON Canada; 6 Department of Family & Community Medicine University of Toronto Toronto, ON Canada

**Keywords:** teleophthalmology, diabetes, diabetic-related retinopathy, screening, primary care, patient, vision loss, screening, Canada

## Abstract

**Background:**

Vision loss from diabetic-related retinopathy (DR) is preventable through regular screening.

**Objective:**

The purpose of this study was to test different patient engagement approaches to expand a teleophthalmology program at a primary care clinic in the city of Toronto, Canada.

**Methods:**

A teleophthalmology program was set up in a large, urban, academic, team-based primary care practice. Patients older than 18 years with type 1 or type 2 diabetes were randomized to one of the following 4 engagement strategies: phone call, mail, mail plus phone call, or usual care. Outreach was conducted by administrative staff within the clinic. The primary outcome was booking an appointment for DR screening.

**Results:**

A total of 23 patients in the phone, 28 in the mail, 32 in the mail plus phone call, and 27 in the control (usual care) group were included in the analysis. After the intervention and after excluding patients who said they were screened, 88% (15/17) of patients in the phone, 11% (2/18) in the mail, and 100% (21/21) in the mail and phone group booked an appointment with the teleophthalmology program compared to 0% (0/12) in the control group. Phoning patients positively predicted patients booking a teleophthalmology appointment (*P*<.001), whereas mailing a letter had no effect.

**Conclusions:**

Patient engagement to book DR screening via teleophthalmology in an urban, academic, team-based primary care practice using telephone calls was much more effective than patient engagement using letters or usual care. Practices that have access to a local DR screening program and have resources for such engagement strategies should consider using them as a means to improve their DR screening rates.

**Trial Registration:**

ClinicalTrials.gov NCT03927859; https://clinicaltrials.gov/ct2/show/NCT03927859

## Introduction

Over 80 million people around the world suffer from diabetic-related retinopathy (DR), the leading cause of blindness for people between the ages of 25 and 74 years [1]. The estimated prevalence of DR among patients with diabetes is 35.4%, and the prevalence is higher among patients with type 1 diabetes, compared to those with type 2 diabetes (77% vs 32%) [2]. Screening every 1-2 years (if there is no pathology) is recommended to prevent the development of DR and blindness [3,4]. Despite this, in Ontario, Canada, more than 400,000 people with diabetes have not been screened in the 2-year period between 2011 and 2013 [5].

Various factors affect the probability of patients getting screened, including environmental factors (eg, accessibility of the clinic, time, and financial concerns), social influences (eg, doctor-patient communication and family influences), knowledge (eg, lack of knowledge about the illness or screening), memory, attention and decision processes (eg, forgetting, absence of symptoms, and competing health issues), beliefs about consequences (eg, perceived necessity and negative short-term effects of the procedure), and emotions (eg, fear, anxiety, and emotional burden) [6]. A recent meta-analysis examining interventions that focused on increasing attendance of DR screening reported that the 2 most commonly used interventions targeting patients were providing “instruction on how to perform the behaviour” and the use of prompts and reminders [7]. Instructions on how to perform the behavior include approaches that provide advice on how often screening should be performed, where one can obtain screening, and how to schedule an exam, whereas approaches with prompts or cues include reminders to perform the behavior, often completed by calling patients or mailing them letters [6]. The review showed that such interventions can increase DR screening attendance by about 12% [7].

Another strategy to improve access to DR screening is a teleophthalmology program [8,9], where images are taken by a trained technician and then sent electronically to an ophthalmologist. The ophthalmologist remotely reviews the images and determines whether the patient needs to be seen in person for further care [10]. Studies have shown that teleophthalmology is a cost-effective alternative to in-person visits performed by eye specialists (ie, optometrists or ophthalmologists) [11,12]. It also has high sensitivity and specificity for the diagnosis of DR, and the diagnostic accuracy is similar to diagnoses provided in clinics [13]. Although teleophthalmology originally emerged as a method to provide access to eye screening for patients living in rural areas, urban teleophthalmology programs have also shown success [9,14-16].

Past studies have demonstrated the effectiveness of patient engagement strategies, such as providing patients with instructions and giving reminders and prompts; however, no studies to date have examined the effectiveness of these methods for engaging patients in teleophthalmology programs. The goal of this study was to evaluate the effectiveness of such patient engagement strategies (eg, calling, mailing letters, or both) in improving diabetes screening care in an urban, team-based primary care setting.

## Methods

### Trial Design

A single-masked randomized factorial design with balanced randomization was used. The trial was registered at ClinicalTrials.gov (NCT03927859) on April 25, 2019.

### Participants’ Eligibility

Patients older than 18 years with a diagnosis of type 1 or 2 diabetes mellitus were eligible to participate in the study. Patients with diabetes were defined as patients whose chart contained an Ontario Health Insurance Plan code K030 (diabetes management assessment) more than once or a problem list containing either “DM,” “dm,” or “diabetes.” Patients who had a record in their chart of having been screened within the last 12 months were excluded from the study.

### Settings

In Ontario, Canada, although the teleophthalmology program has been in practice for almost 20 years in rural regions, the urban program has been in practice since 2013 and was developed in response to the finding that many underscreened individuals live in urban areas [5]. The program was initially set up through community health centers but started expanding to other primary care settings outside community health centers. With the goal to expand the program to more settings outside community health centers, the program was introduced to an urban, academic, team-based primary care practice. The urban program in Toronto, Ontario is currently deployed at 11 core sites across the province [17]. Staff from each core site carry equipment to various satellite locations. Together with the core sites, the program provides services to 74 sites across Ontario. The focus of the program is to provide screening services for underserved and vulnerable groups and regions. Patients can be referred to the program either by a primary care physician, a nurse practitioner, or a diabetes education program personnel. The cost of delivery is covered by the publicly funded health care system in Ontario (ie, Ontario Health Insurance Plan). The study was conducted at one of the satellite locations of the Toronto, Ontario teleophthalmology program—Women’s College Hospital Family Health Team—a hospital-affiliated team-based primary care practice. Family Health Teams in Ontario, Canada consist of a team of family physicians and nurse practitioners, supported by registered nurses, social workers, dietitians, and other professionals. The practice had recently (within a month) become a satellite site for teleophthalmology, meaning that a nurse was available once a week to screen patients locally.

### Ethics Approval

The study was approved by the Women’s College Hospital research ethics board (2018-0068) through delegated review.

### Consent to Participate

All methods were performed in accordance with the Canadian guidelines and regulations (ie, the Tri-Council Policy Statement: Ethical Conduct for Research Involving Humans—TCPS 2). Individual consent from patients was waived by the research ethics board prior to the intervention, as the study posed a minimal risk to patients, it was run entirely within the primary care practice, and informing patients about the purpose of the study (ie, increasing diabetes eye screening rates) ahead of the intervention would have contaminated the results. No identifiable information on patients was provided to the external research team. All patients were asked at the end of the trial, during the last contact with patients, whether they consented to sharing their information for research purposes. Patients who refused and those who were not asked for consent for various reasons (eg, they wanted to discontinue the call) were excluded from the study (70/182, 38% of the sample).

### Interventions

Patients were randomized to one of the following 4 intervention groups: phone call, mail, mail plus phone call, and usual care. Patients assigned to the phone call intervention were contacted by an administrative staff who informed them that they were calling from the family practice and asked them whether they have had their diabetes eye screening exam completed in the recent year. Patients who indicated that they were not screened and did not have a preexisting scheduled appointment were offered to make a booking for the teleophthalmology program and be screened at the family practice. Patients who were assigned to the mailed letter intervention received a letter in the mail from the practice stating that they were due for DR screening, and the letter provided them with a list of options for screening, such as receiving a referral for ophthalmology from their family doctor, going to an optometrist’s office, or visiting the teleophthalmology program at their clinic. The letter also included a brochure about the teleophthalmology program. Patients in the letter plus phone call group were sent out the letter and then were contacted by phone a week later. The usual care group was called a month after the trial was initiated, and patients were asked whether they had been screened to obtain a baseline screening rate for them. Usual care at this practice consisted of primary care providers asking patients during a routine visit whether they had been screened for DR within the last year and providing a referral to an optometrist, an ophthalmologist, or the local teleophthalmology program for screening. When referral was made, patients would receive a call with the date of their appointment from the primary care practice.

### Randomization

In total, 4 physicians with the largest numbers of patients with diabetes mellitus at the practice and 1 physician with just a few patients participated in the study. The rest of the physicians in this practice were allocated to a concurrent physician engagement study that required a larger number of physicians and were, as a result, excluded from the trial. The 5 participating physicians initially had a total of 215 eligible patients with diabetes, but upon closer review of the list, only 182 patients met the inclusion criteria to be randomized. Once a comprehensive list of patients with diabetes across the rosters of these 5 physicians was collected, they were allocated to groups by matching the list of names to a randomly created sequence of group allocations that were created on the randomizer.org website. The list contained a random sequence of one of the 4 groups in a 1:1:1:1 ratio.

### Outcomes

The primary outcome was the total number of patients who made a booking with teleophthalmology. This was assessed at the time of the call for interventions that involved a call; for the mailed letter group, these data were collected by making a phone call a month after the letters were sent out.

### Sample Size

A recent systematic review of quality improvement interventions targeting DR screening concluded that the interventions result in a risk difference of 17% on average [7]. Using this information, we assumed that if 50% of the patients at baseline were to be screened, then 67% of patients in the intervention groups would be screened or booked for screening post intervention. The resulting required sample size was 210 participants with α=.05, 1-tailed, to achieve a power of 0.80.

### Masking

This was a single-masked trial. Patients were not aware that they were part of a study until the end of the call or until the time they were contacted (for the mail only group and the control group).

### Statistical Methods

We ran a linear model logistic regression with group assignments as the predictor variables and whether a patient made a booking with the teleophthalmology program as the outcome variable.

## Results

### Participant Flow

**Figure 1:**
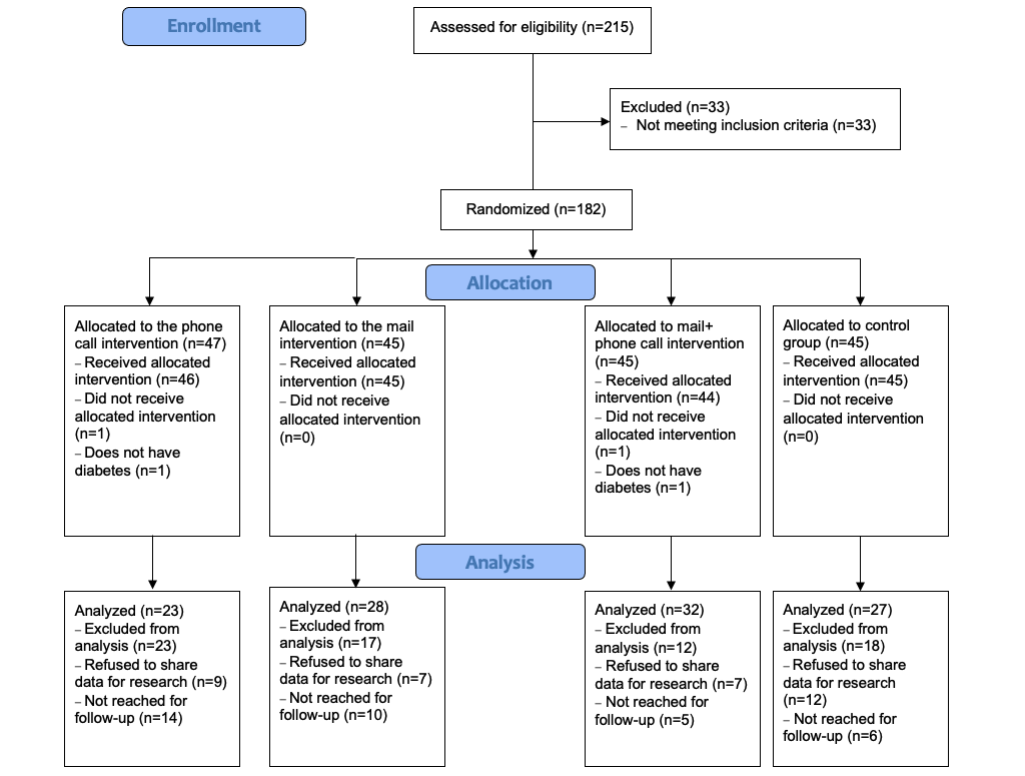
Flow diagram showing patients flow through the study.

A total of 182 patients were randomized to one of the 4 groups using randomizer.org website to generate numbers for each group allocation; 47 patients were allocated to the phone call intervention, and 45 each were allocated to the mail intervention, the mail and phone intervention, and the usual care group. One patient in the phone and one in the mail and phone group did not receive the intervention (Figure 1). The random allocation sequence was generated by VS, and enrollment and assignment of participants was done by an administrative assistant at the clinic. Patient characteristics per group are displayed in Table 1.

**Table 1:** Patient characteristics per group.

Characteristics		Phone (n=23)	Mail (n=28)	Phone and mail (n=32)	Control (n=27)
**Sex, n (%)**					
	Female	10 (43)	16 (57)	20 (63)	10 (37)
	Male	13 (57)	12 (43)	12 (37)	17 (63)
Age (years), mean (SD)		66 (15)	63 (15)	69 (14)	65 (14)
**Diabetes type**					
	Type 1	1 (4)	1 (4)	4 (13)	4 (15)
	Type 2	15 (65)	21 (75)	22 (69)	13 (48)
	Type unknown	7 (30)	6 (22)	6 (19)	10 (37)
PCP^a^ visits per year, mean (SD)		22 (13)	20 (15)	28 (16)	22 (14)
Patients screened elsewhere before the intervention^b^		6 (26)	10 (36)	11 (34)	15 (56)

^a^PCP: primary care physician.

^b^Patients who indicated they were screened elsewhere when we contacted them. Initially, only patients who were unscreened based on their medical record were included in the patient groups, but when we spoke to patients, some patients indicated they had already been screened elsewhere; however, we were not aware of it, as it had not been recorded in their medical record.

### Recruitment

The intervention for the trial began in July 2019 and was completed in September 2019.

### Numbers Analyzed

After some patients refused to share their data for research and others were not reached for follow-up, a remainder of 23 patients in the phone, 28 in the mail, 32 in the mail and phone, and 27 in the control were included in the analysis (Table 1; Figure 1).

### Intervention Results

We first excluded patients who indicated that they were already screened or already have a preexisting appointment, as these patients were not eligible for screening. After the intervention, 88% (15/17) of patients in the phone intervention, 11% (2/18) of patients in the mail group, and 100% (21/21) in the mail and phone group booked an appointment with the teleophthalmology program compared to none in the Control Group (0/12). We ran a logistic regression model with whether a patient booked an appointment with the teleophthalmology program as the dependent variable and the types of intervention received (ie, mail, phone, or mail and phone) as the predictor factors. This analysis was done only on patients who were not screened at the time of intervention. As all patients in the mail and phone group booked an appointment, and none in the control had booked an appointment, the regression analysis could not be completed due to complete separation. We therefore ran one logistic regression model looking at the mail intervention and another model looking at the effect of the phone intervention. The logistic regression looking at the phone intervention showed that phoning patients positively predicted patients booking a teleophthalmology appointment (*P*<.001; odds ratio=252, 95% CI 33.3 to >999). The logistic regression looking at the effect of sending a letter showed no effect (*P*=.55; odds ratio=1.3, 95% CI 0.5-3.5). Despite not being able to include the interaction effect in the analysis, we should report that none of the patients in the mail and phone group booked an appointment in response to the letter, and all of them booked an appointment at the time they received the phone call.

## Discussion

### Principal Findings

In this randomized factorial design study, we compared the effectiveness of the use of phone call, mailed letter, as well as mail and phone call combined for engaging patients into booking an appointment for a primary care practice embedded with a teleophthalmology program. We found that calling patients was much more effective than sending a letter; we also found that sending a letter ahead of the phone call did not further increase the likelihood of booking an appointment.

Although no studies have examined the effectiveness of these patient engagement approaches in the context of a teleophthalmology program, many have examined their effectiveness in the context of regular in-person screening programs for DR. Our findings are consistent with previous studies examining improving engagement in DR screening, showing that phoning patients is more effective than mailing out letters [18-20], with one study reporting a 74% increase in retinopathy screening in the telephone versus the information mail-out group [19]. Similar findings have been reported in studies trying to engage patients in other screening procedures within primary care settings [21]. Although making phone calls is more costly than sending letters [18,22], our study suggests that despite its lower cost, mailing letters to patients has a very low effect on engaging patients in a teleophthalmology program that is embedded in a team-based primary care practice. A phone call may allow patients to ask questions and book an appointment on the spot, combining education with convenience. A stronger educational component may also boost the effectiveness of letters. For example, one study showed that simple reminder letters combined with an automated phone reminder are not as effective as a mailed out educational brochure combined with a personalized letter [23]. Other studies have suggested that the greater effectiveness of phone calls lies in the ability to personalize the engagement approach [20].

### Implications for Primary Care Practice

This study was conducted in a team-based practice that has administrative staff who can contact patients and engage them in DR screening. Practices that do not have the administrative resources to engage patients through phone calls may require physicians to find alternative ways to engage patients. One potential solution could be to engage with local teleophthalmology programs in a partnership, so that unscreened patients with diabetes in their practice can be contacted by the teleophthalmology program directly. It is important to note that due to the preexisting relationship between primary care providers and patients, screening engagement has been shown to be more effective if patients are being contacted by their primary care provider rather than by an independent program [24,25]. Therefore, it is important for independent programs to work directly with primary care practices, so that patients can be assured that their own provider has been involved in the decision to ask them to be screened. A qualitative study examining motivators behind engaging in a teleophthalmology program in an urban setting suggested that patients especially value recommendations coming from their own primary care provider [26].

### Strengths and Limitations

The strengths of this study include the randomized factorial design and the pragmatic implementation, where interventions were fully managed by administrative staff at the primary care practice, both of which increase external validity. Our study also has several limitations. First, this was a study that was conducted within the context of a large, academic, team-based primary care practice with external funding to pay for additional time for a casual staff member. This would be more difficult to execute without additional administrative support in smaller settings. Second, our interventions (ie, letter or phone call) limit our sample to patients who have a phone and an address, speak English, and have the literacy required for being able to read. As such, generalizability of our findings is limited to these patient populations. Third, we had to exclude a subset of providers from the study due to another concurrent physician trial being conducted; many patients had been screened already at the time they were contacted, and some asked their data to be excluded from analyses; as a result, our final sample size was much smaller than initially planned; nonetheless, the results with regard to the effectiveness of phone calls are quite evident. Our results suggested that phone calls are likely to be superior to letters, but we were unable to examine the effect of the interaction between phone call and letters, as all the patients assigned to the phone call and letter group booked an appointment. A larger sample size is needed to confirm the effects of that interaction. Finally, our primary outcome was booking of an appointment, and we were unable to link attendance to the screening appointment or attendance of screenings outside the teleophthalmology program. Many patients were booked for screening several months after the time of booking, and the project was set to be completed before the time of bookings; therefore, we were not able to ascertain exactly which patients eventually attended the booking. We do know from communication with the program, however, that 78% of the patients who booked a teleophthalmology appointment with the physicians whose patients participated in this trial have shown up for their teleophthalmology appointment.

This study demonstrated that phone calls were highly effective in recruiting patients to an urban teleophthalmology program, but this intervention may be difficult to scale without external funding due to capacity constraints in primary care. Future studies could focus on regional support for different methods of directly reaching out to patients, such as letters with a stronger educational component, personalized letters, emails, and text messages. Having the ability to link health administrative data that verify whether the patient has been screened and providing primary care practices with a list of patients who have not been screened is also another approach that would enable practices to more accurately target patients who have not been screened.

## Abbreviations

DR: diabetic-related retinopathy

## Data Availability

The data sets generated and analyzed during this study are not publicly available due to data sharing restrictions but are available from the corresponding author on reasonable request.
